# Time to Treatment Intensification in Patients Receiving DPP4 Inhibitors *Versus* Sulfonylureas as the First Add-On to Metformin Monotherapy: A Retrospective Cohort Study

**DOI:** 10.3389/fphar.2022.871052

**Published:** 2022-05-30

**Authors:** Giuseppe Roberto, Anna Girardi, Francesco Barone-Adesi, Alessandro Pecere, Valentina Ientile, Claudia Bartolini, Roberto Da Cas, Stefania Spila-Alegiani, Carmen Ferrajolo, Paolo Francesconi, Gianluca Trifirò, Elisabetta Poluzzi, Fabio Baccetti, Rosa Gini

**Affiliations:** ^1^ Osservatorio di Epidemiologia, Agenzia Regionale di Sanità Della Toscana, Firenze, Italy; ^2^ Dipartimento di Medicina Traslazionale, Università Del Piemonte Orientale, Novara, Italy; ^3^ Dipartimento di Scienze Biomediche, Odontoiatriche e Delle Immagini Morfologiche e Funzionali, Università Degli Studi di Messina, Messina, Italy; ^4^ Centro Nazionale per la Ricerca e la Valutazione Preclinica e Clinica Dei Farmaci, Istituto Superiore di Sanità, Roma, Italy; ^5^ Dipartimento di Medicina Sperimentale, Università Degli Studi Della Campania “L. Vanvitelli” e Centro Regionale di Farmacovigilanza, Regione Campania, Napoli, Italy; ^6^ Unità di Farmacologia, Dipartimento di Scienze Mediche e Chirurgiche, Università di Bologna, Bologna, Italy; ^7^ Unità Operativa di Diabetologia Massa-Carrara, USL Toscana Nordovest, Massa, Italy

**Keywords:** type 2 diabetes, DPP4i, sulfonylurea, metformin, treatment intensification, durability, secondary failure, observational study

## Abstract

**Background:** To verify whether, in patients on metformin (MET) monotherapy for type 2 diabetes (T2D), the add-on of a dipeptidyl peptidase inhibitor (DPP4i) compared to a sulfonylurea (SU) can delay the time to the subsequent treatment intensification (TI).

**Methods:** Population-based administrative data banks from four Italian geographic areas were used. Patients aged ≥18 years on MET monotherapy receiving first DPP4i or SU dispensing between 2008 and 2015 (cohort entry) were followed up to the occurrence of TI (insulin dispensing or add-on of a third non-insulin hypoglicemic >180 days after cohort entry), treatment discontinuation, switch, cancer, death, TI occurrence within, end of data availability, end of study period (31 December 2016), whichever came first. Patients on MET + DPP4i were matched 1:1 with those on MET + SU by sex, age, year of cohort entry, and data bank. Hazard Ratio (HR) and 95% confidence intervals (95%CI) were estimated using multivariable Cox regression model including matching variables and potential confounders measured at baseline. Different sensitivity analyses were performed: i) matching at 180 days after cohort entry, ii) intent to treat (ITT) analysis, iii) matching by duration of MET monotherapy, iv) matching by propensity score.

**Results:** The matched study cohort included 10,600 patients. Overall, 763 TI were observed (4.5/100 person-years; mean follow-up = 1.6 years). The primary analysis showed no difference in time to TI between the two groups (HR = 1.02; 95% CI = 0.88–1.19). Sensitivity analyses confirmed this result, except from the ITT analysis (HR = 1.27; 1.13–1.43).

**Conclusion:** The use of a DPP4i rather than a SU as add-on to MET monotherapy was not associated with a delay in treatment intensification.

## Introduction

Diabetes is a chronic metabolic condition causing sustained hyperglycemia due to a deficit of insulin secretion and/or a reduced response of target tissues to this hormone (Merckmanuals, 2021). Type 2 diabetes (T2D), in which insulin-resistance is the predominant pathogenetic mechanism, represents about the 90% of all diabetes cases worldwide (Alberti and Zimmet, 1998). Chronic exposure to hyperglycemia can cause the occurrence of serious and potentially fatal micro- and macrovascular complications (Merckmanuals, 2021). Therefore, patients with T2D are strongly recommended to start a hypoglycemic medication whenever diet and life style modification are not sufficient for maintaining glycemic control (Merckmanuals, 2021; Italian Standards of Medical Care of Diabetes, 2014; American Diabetes Association, 2015).

Metformin is generally considered as the first choice for the initial treatment of T2D (Italian Standards of Medical Care of Diabetes, 2014; Montilla et al., 2014). However, due the progressive nature of the disease, hypoglycemic drugs tend to lose their efficacy over time (i.e., secondary treatment failure) so that treatment intensification might be necessary to maintain the recommended glycemic target (Drucker and Nauck, 2006; Pitocco et al., 2008; White, 2009; Zheng et al., 2018; Kalra et al., 2019).

In addition to traditional second-line non-insulin hypoglycemic drugs such as sulfonylureas, glinides, glitazones, and acarbose, in February 2008 the Italian Healthcare Service approved the reimbursement of the first incretin-based medicines (Azoulay, 2015). The clinical efficacy of this class of drugs in the treatment of T2D relies on the potentiation of the activity of the Glucagon-like peptide 1 (GLP-1), an endogenous hormone belonging to the family of incretin hormones that exerts an important role in the glycemic homeostasis (Schneeweiss et al., 2011). Currently available incretin-based medicines are distinguished in two main groups: GLP-1 analogues (GLP1a) and dipeptidyl peptidase-4 inhibitors (DPP4i). Indeed, DPP4i are the most widely used incretin-based therapies, given their higher convenience of use compared to GLP1a (i.e., oral vs. subcutaneous administration) (Schneeweiss et al., 2011; Italian Standards of Medical Care of Diabetes, 2014; Roberto et al., 2019).

Results from clinical trials have suggested a positive risk/benefit balance of DPP4i in the treatment of T2D (Moride et al., 2005; Schneeweiss et al., 2011). Moreover, results from pre-clinical studies showed a favorable effect on b cell preservation (Deacon, 2004; Drucker and Nauck, 2006). In fact, other than stimulating glucose-dependent insulin secretion, activation of the GLP-1 receptor was found to be associated with increased b cell proliferation and inhibition of b cell apoptosis in different *in vivo* (Edvell and Lindström, 1999; Pospisilik et al., 2002; Pospisilik et al., 2003) and *in vitro* studies (Farilla et al., 2003; Wang et al., 2004). For these reasons, a potential advantage of DPP4i in terms of treatment durability (i.e., time to secondary treatment failure) compared to other hypoglycemic agents was hypothesized (Drucker and Nauck, 2006). However, currently available clinical evidence on DPP4i treatment durability is still scarce and inconclusive (Schneeweiss et al., 2011; Pottegård et al., 2014; Mishriky et al., 2015; Rafaniello et al., 2015; Deacon and Lebovitz, 2016; Foroutan et al., 2016; Mamza et al., 2016; Moreno Juste et al., 2019; Chen et al., 2017). Shedding light on this fundamental aspect of T2D pharmacotherapy can help to better establish the place in therapy of DPP4i compared to other widely used second-line oral hypoglycemic agents such as sulfonylureas (SU) (Mishriky et al., 2015; Deacon and Lebovitz, 2016; Foroutan et al., 2016; Moreno Juste et al., 2019) and have significant impact on drug policies and prescribing recommendations.

Therefore, the aim of this study was to analyse routinely collected administrative data from four Italian geographic areas to verify whether, among patients on metformin (MET) monotherapy for T2D, the add-on of a DPP4i compared to SU was associated with a delay in treatment intensification, which was considered as a proxy of secondary treatment failure.

## Materials and Methods

### Data Source

Italy has a tax-based, universal coverage National Health System organised in three levels: national; regional (21 regions); and local (on average, 10 Local Health Authorities per region). Healthcare is managed, for every inhabitant by the relevant Local Health Authority (LHA) (Trifirò et al., 2019).

This study was based on the analysis of data from four Italian regions, Piedmont (northern Italy), Tuscany and Umbria (central Italy), and one LHA, Caserta (southern Italy) covering an overall source population of around 10 million people (http://demo.istat.it/bil2015/index.html). The four data sources are based on different data banks (Thurin et al., 2021), which collect person-level information on the utilization of healthcare services reimbursed by the National Healthcare Service (NHS) and dispensed to any subject who is resident and registered with a general practitioner in the relevant catchment areas. Through a pseudoanonymized identification code, patient-level information recorded in different registries can be linked. For the purposes of this study, data from the following five data banks were used: 1) inhabitant registry, 2) hospital discharge records, 3) drug registry, 4) reason for exemption from copayment registry, and 5) registry of utilization of secondary care encounters and diagnostic procedures. The drug registry includes dispensing of prescription drugs intended for outpatient use (e.g., dispensing date, active principle, ATC code, brand name and formulation). The hospital discharge record registry contains information on hospitalization episodes (e.g., date of admission/discharge, discharge diagnoses and procedures code with ICD9-CM terminology). The exemption from copayment registry includes information on the disease that allows patients to be exempt from copayment of a specific list of healthcare services. The registry of secondary care and diagnostic activities include information on the utilization of specialist outpatient encounters, diagnostic tests or procedures (e.g., date, type of specialist visit, test or procedure), but not the results of tests or the diagnosis of the patient. Given the administrative nature of the data source, records are only accepted in the system if all relevant field are correctly filled out.

### Selection of the Study Cohort

Patients in the study areas with ≥1 dispensing of a DPP4i or SU (see Supplementary Appendix S1 for ATC codes) recorded between first of February 2008 and 30 June 2015 were identified (due to difference in data availability, the start date of the recruitment period differed depending on the specific area, see Supplementary Appendix S2). The date of the first dispensing of a DPP4i or SU (index prescription) was the cohort entry. Patients aged <18 and with a look-back period <1 year were excluded. To select patients that received a DPP4i or a SU as first add-on to metformin monotherapy, only individuals with ≥1 metformin dispensing recorded at least 60 days before cohort entry were retained in the study cohort (Hayes et al., 2006; Ema, 2021b) (Supplementary Figure 1). Moreover, patients had to be persistent to metformin monotherapy (see below for the definition of persistence), and without any record of antidiabetic drug dispensing other than metformin (see Supplementary Appendix S1) during the year preceding the index prescription. Patients with a cancer diagnosis (ICD9CM codes: 140–239) recorded at any time before the index prescription were also excluded.

On the basis of the add-on treatment received at cohort entry, patients were classified in the relevant treatment group, i.e., MET + DDP4i or MET + SU.

### Study Design and Exposure Definition

This was a retrospective cohort study. Patients in the two groups were followed starting from the index dispensing up to the occurrence of either the study outcome (i.e., treatment intensification) or a censoring event, whichever came first. Events that were considered as censoring criteria were: non-persistence to metformin, non-persistence to the index drug, switch to a different non-insulin hypoglycemic medication (see Supplementary Appendix S3 for description of the operational definitions of these events), end of study period (31 December 2016), cancer, death, or emigration from the region/LHU of recruitment.

Treatment persistence was defined as the absence of any gap ≥90 days between the end of the estimated duration of a dispensing and the subsequent dispensing date (Greevy et al., 2011). The duration of each observed dispensing was calculated by using the relevant Defined Daily Dose (https://www.whocc.no/atc_ddd_index/).

Each patient on MET + DPP4i treatment was 1:1 matched to patients in the MET + SU treatment group. Matching was performed by age band category (18–44, 45–54, 55–64, 65–74, 75–84, 85 + ), sex, calendar year of index prescription and geographical area.

### Variables at Baseline

The following variables were measured at baseline (index prescription): age, sex, calendar year of cohort entry, number of encounters with a diabetologist recorded during the year before index prescription. The time elapsed between the first metformin dispensing and the index dispensing (either DPP4i or SU) was used as a proxy of disease duration. For the purpose of sensitivity analyses (see below), this time was also classified either as “definite”, for patients with ≥1 year of observation before the first observed metformin dispensing, or “uncertain” (see Supplymentary Appendix S4).

Diabetes complications and comorbidities were measured at baseline through diagnoses recorded, either at hospital discharge or as an exemption from copayment, during the year preceding the index prescription (see Supplementary Appendix S5).

Similarly, we also measured the use of medications that might affect glycemic control during the year preceding the index prescription (antidepressants, antipsychotics, corticosteroids for systemic use, lipid-lowering drugs, low-dose aspirin, antihypertensive, thiazides, statins, beta-blockers—see Supplementary Appendix S6).

### Outcomes

The primary outcome was the occurrence of treatment intensification, defined as either the initiation of insulin treatment (first dispensing of insulin) or the add-on of a third non-insulin antidiabetic (see Supplymentary Appendix S3 for details) (Ema, 2021a; Drucker and Nauck, 2006; Greevy et al., 2011; Anichini et al., 2013; Inzucchi et al., 2015a; Gini et al., 2016). Differently from primary treatment failure, secondary treatment failure occurs when glycemic control is lost after an initial period during which the pharmacological treatment was effective in achieving glycemic control (Pitocco et al., 2008) Since administrative data used for this study do not provide information on glycemic level, distinction between primary treatment failures and early secondary treatment failure was not possible. Therefore, similarly to other previously performed observational studies (Brown et al., 2010), all treatment intensifications occurred during the first 180 days, which are likely to mostly correspond to primary treatment failure, were censored to avoid outcome misclassification.

### Statistical Analysis

Survival curves describing the time to treatment intensification in the matched cohort were plotted using the Kaplan-Meier method and the log rank test was used to assess the statistical significance of the difference between groups.

Cox regression models were applied to estimate hazard ratios, with their 95% confidence intervals, and compare the time to treatment intensification from index prescription in patients treated with MET + DPP4i vs. those in the MET + SU group. All the variables measured at baseline were included in the model to account for their potential confounding effect.

### Sensitivity Analyses

In order to evaluate the robustness of our results, we carried out different sensitivity analyses: 1) a Propensity Score-matched analysis with caliper width of 0.1 was performed (Farr et al., 2014). Variables considered for PS included all patients’ characteristics measured at baseline. 2) Since disease duration is an important predictor of the durability of the hypoglycemic efficacy of antidiabetic drugs (Wilke et al., 2016), the primary analysis was re-run restricting the study cohort to patients with “definite” time between first antidiabetic dispensing and index drug. 3) Since a significant imbalance in treatment discontinuation probability was observed between the two treatment groups, particularly during the first 6 months from cohort entry (data not shown), start of follow-up time was set at 180 days after index prescription. 4) Finally, an intent-to-treat (ITT) approach was used, in which we did not censored neither for discontinuation nor for switch.

### Data Management and Analysis

In order to standardize the process of data extraction and management, each study partners run the open-source software TheMatrix (http://thematrix.isti.cnr.it/) locally. As a result, an aggregated analytical dataset was obtained and shared with all the study participants only after local partner’s verification and approval. The Regional Agency for Healthcare Services of Tuscany was responsible for the analyses of the shared analytical dataset. These were performed with the statistical software STATA (version 14).

The full protocol of this study was published in advance to data extraction and analysis on the ENCePP EU PASS Register (freely available at: https://www.encepp.eu/encepp/viewResource.htm?id=28096).

## Results

**FIGURE 1:**
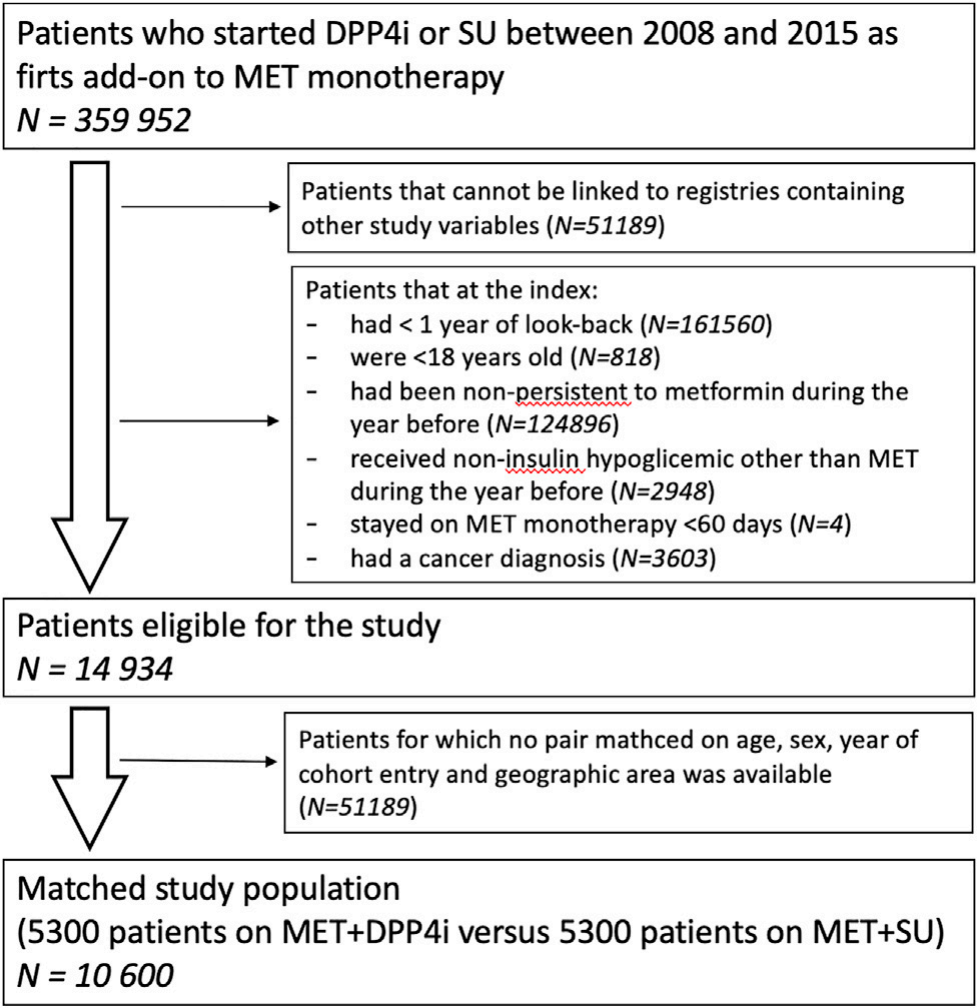
Selection of the study population.

A total of 14,934 patients that received at least one DPP4i or SU dispensing as add-on to prior metformin monotherapy were identified (Figure 1). Among them, 6,261 (42%) patients were in treatment with MET + DPP4i, while 8,673 (58%) with MET + SU (Table 1). Most of the patients identified were from Tuscany (44.1%) and Piedmont (32.7) (Supplementary Table 1). After 1:1 matching by age at index prescription, sex, calendar year of index prescription and geographical area, a cohort of 10,600 patients was included in the analysis (5,300 patients in each group). Overall, most of the patients in the matched study cohort were male (57.2%) and the great majority (81.5%) of the enrolled patients were aged ≥55 years (Table 1). Patients in treatment with MET + SU compared with MET + DDP4i users, differed in utilization of some medications, e.g., systemic corticosteroids (MET + iDPP4 = 11.5% vs. MET + SU = 14%) and lipid lowering medications (MET + iDPP4 = 63% vs. MET + SU = 55%).

**TABLE 1:** Cohort characteristics before and after matching.

	Pre-matching		Post-matching	
	DPP4i (6,261)	SU (8,673)	DPP4i (5,300)	SU (5,300)
	**N (%)**	**N (%)**	**N (%)**	**N (%)**
Women	2,609 (41.7)	4,110 (47.4)	2,268 (42.8)	2,268 (42.8)
Age band				
18–44	275 (4.4)	297 (3.4)	204 (3.8)	204 (3.8)
45–54	1,080 (17.2)	1,013 (11.7)	775 (14.6)	775 (14.6)
55–64	2,103 (33.6)	2,259 (26.1)	1712 (32.3)	1712 (32.3)
65–74	1930 (30.8)	2,921 (33.7)	1773 (33.4)	1773 (33.4)
75–84	774 (12.4)	1831 (21.1)	748 (14.1)	748 (14.1)
85+	99 (1.6)	352 (4.1)	88 (1.7)	88 (1.7)
Cohort entry				
2008	82 (1.3)	755 (8.7)	81 (1.5)	81 (1.5)
2009	187 (3.0)	767 (8.8)	186 (3.5)	186 (3.5)
2010	338 (5.4)	802 (9.3)	338 (6.4)	338 (6.4)
2011	706 (11.3)	738 (8.5)	559 (10.6)	559 (10.6)
2012	933 (14.9)	804 (9.3)	695 (13.1)	695 (13.1)
2013	1715 (27.4)	1883 (21.7)	1,416 (26.7)	1,416 (26.7)
2014	1,282 (20.5)	1974 (22.8)	1,222 (23.1)	1,222 (23.1)
2015	1,018 (16.3)	950 (10.9)	803 (15.1)	803 (15.1)
Time since 1st MET				
0	283 (4.5)	410 (4.7)	188 (3.6)	252 (4.8)
1	1,012 (16.2)	1,309 (15.1)	768 (14.5)	815 (15.4)
2	902 (14.4)	1,342 (15.5)	746 (14.1)	826 (15.9)
3	1,388 (22.2)	1791 (20.7)	1,218 (23.0)	1,156 (21.8)
4+	2,676 (42.7)	3,821 (44.1)	2,380 (44.9)	2,251 (42.5)
Diabetes-related comorbidities				
Acute myocardial infarction	33 (0.5)	79 (0.9)	32 (0.6)	48 (0.9)
Acute ischemic heart disease	37 (0.6)	50 (0.6)	33 (0.6)	22 (0.4)
Angina pectoris	23 (0.4)	41 (0.5)	21 (0.4)	25 (0.5)
Operations on vessel of heart	72 (1.2)	111 (1.3)	65 (1.2)	64 (1.2)
Cerebrovascular diseases	27 (0.4)	64 (0.7)	24 (0.5)	34 (0.6)
Retinopathy	4 (0.1)	3 (<0.0)	3 (0.1)	1 (<0.0)
Diabetes with ophthalmic manifestations	5 (0.1)	1 (<0.0)	4 (0.1)	1 (<0.0)
Diabetes with renal manifestations	10 (0.2)	8 (0.1)	8 (0.2)	4 (0.1)
Acute kidney failure	11 (0.2)	14 (0.2)	11 (0.2)	5 (0.1)
Diabetes with peripheral circulatory disorders	38 (0.6)	65 (0.8)	36 (0.7)	42 (0.8)
Ulcer of lower limbs, except pressure ulcer	5 (0.0)	8 (0.1)	5 (0.1)	6 (0.1)
Concomitant Pharmacotherapies				
Antidepressants	815 (13.0)	1,468 (16.9)	706 (13.3)	842 (15.9)
Corticosteroids for systemic use	699 (11.2)	1,249 (14.4)	607 (11.5)	740 (14.0)
Lipid lowering drugs	3,897 (62.3)	4,658 (53.7)	3,355 (63.3)	2,921 (55.1)
Anticoagulants	656 (10.5)	1,101 (12.7)	581 (11.0)	604 (11.4)
Antiplatelets	2,497 (39.9)	3,626 (41.8)	2,187 (41.3)	2,100 (39.6)
Beta blockers	2009 (32.1)	2,781 (32.1)	1719 (32.4)	1708 (32.2)
Antihypertensives and/or diuretics	1,379 (22.0)	2,442 (28.2)	1,207 (22.8)	1,331 (25.1)
Dihydropyridine CCB	1,407 (22.5)	2,116 (24.4)	1,208 (22.8)	1,244 (23.5)
Non Dihydropyridine CCB	158 (2.5)	247 (2.9)	142 (2.7)	130 (2.5)
Angiotensin receptor blockers and ACE-I	4,207 (67.2)	5,859 (67.6)	3,596 (67.9)	3,521 (66.4)
Antipsychotics	139 (2.2)	304 (3.5	110 (2.1)	193 (3.6)

MET, metformin; DDP4i = dipeptidyl peptidase inhibitor; SU, sulfonylurea.

The average available time of observation time for patients in the cohort was about 4 years and a half, however the application of the censoring criteria resulted in a mean follow-up time of 1.9 years for patients in treatment with MET + DDP4i and 1.2 years for those treated with MET + SU. The main causes of censoring were related to discontinuation of either the index drug or MET, with a more frequent occurrence for patients in treatment with MET + SU (overall 76.5%) compared to MET + DD4i (overall 66.8%) (Supplementary Table 2).

**FIGURE 2:**
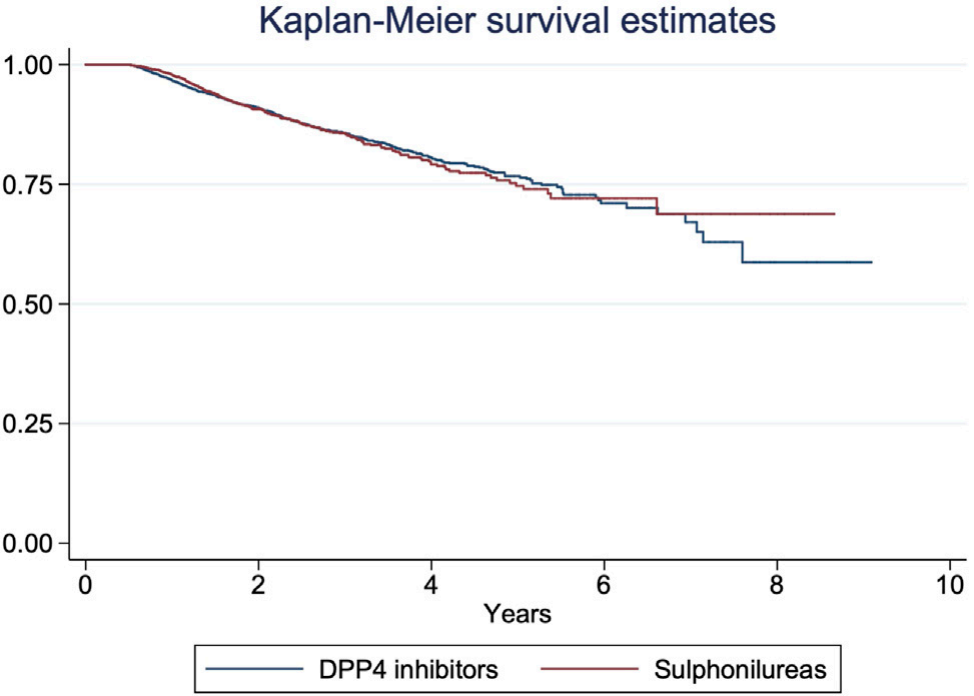
Kaplan-Meier survival estimate in the matched study cohort.

A total of 763 treatment intensification was observed, corresponding to an incidence rate of 4.5 per 100 person-years. Kaplan-Meier survival curve describing time to treatment intensification showed no significant differences (*p* = 0.89) in time to treatment intensification between the two matched groups (Figure 2). Cox regression yielded comparable results to those obtained with the Kaplan-Meier method (Table 2) showing no significant differences between the two groups in terms of time to treatment intensification (HR: 1.02; 95%CI:0.88–1.19). The regression analysis also showed that patients aged 55–84 years had a lower risk for treatment intensification compared to younger patients aged 18–44 years (Table 2). Moreover, the risk of treatment intensification appeared to increase along with the time from first metformin dispensing. A positive association with treatment intensification was also observed in patients using antidepressants (adj HR: 1.25; 95%CI: 1.02–1.54) and antihypertensive drugs (adj HR: 1.28; 95%CI: 1.07–1.54) compared to non-users. Finally, patients from Piedmont and Umbria, respectively were less likely to receive a treatment intensification compared to those from Caserta.

**TABLE 2:** Results from the multivariate Cox regression model.

	adj HR*	[95%CI]	
MET + iDPP4	Ref	Ref	Ref
MET + SU	1.02	0.88	1.19
Men	Ref	Ref	Ref
Women	0.93	0.80	1.08
Age band			
18–44	Ref	Ref	Ref
45–54	0.86	0.61	1.22
55–64	0.57	0.41	0.80
65–74	0.45	0.32	0.64
75–84	0.43	0.28	0.64
85+	0.74	0.37	1.48
Cohort entry			
2008	Ref	Ref	Ref
2009	1.50	0.83	2.69
2010	1.16	0.66	2.04
2011	1.22	0.71	2.11
2012	1.13	0.65	1.95
2013	0.93	0.53	1.62
2014	0.90	0.51	1.57
2015	0.61	0.33	1.11
Geographical area			
Caserta	Ref	Ref	Ref
Piemonte	1.38	1.03	1.87
Toscana	1.02	0.79	1.32
Umbria	1.73	1.25	2.41
Time since 1st MET			
0	Ref	Ref	Ref
1	1.34	0.86	2.09
2	1.54	0.98	2.40
3	1.62	1.04	2.52
4+	1.66	1.08	2.57
Diabetes-related comorbidities			
Acute myocardial infarction	0.65	0.14	3.08
Acute ischemic heart disease	1.27	0.31	5.25
Angina pectoris	1.65	0.59	4.64
Cerebrovascular diseases	1.38	0.50	3.78
Diabetes with neurological manifestations	2.94	0.72	12.08
Diabetes with peripheral circulatory disorders	0.24	0.03	1.77
Operations on vessel of heart	0.69	0.22	2.13
Concomitant pharmacotherapies			
Antidepressants	1.25	1.02	1.54
Corticosteroids for systemic use	0.95	0.75	1.21
Lipid lowering drugs	0.95	0.82	1.11
Anticoagulants	1.02	0.79	1.32
Antiplatelets	1.10	0.93	1.30
Beta blockers	1.08	0.92	1.28
Antihypertensives and/or diuretics	1.28	1.07	1.54
Dihydropyridine CCB	1.05	0.88	1.26
Non Dihydropyridine CCB	1.46	0.96	2.22
Angiotensin receptor blockers and ACE-I	1.00	0.85	1.17
Antipsychotics	1.26	0.83	1.90

*adjusted hazard ratio for all covariates measured at baseline.

MET, metformin; DDP4i = dipeptidyl peptidase inhibitor; SU, sulfonylurea.

Overall, results from the sensitivity analyses (Table 3) were in line with those from the primary analyses and did not highlighted differences in rate of treatment intensification in patients treated with MET + DPP4i vs. MET + SU, with the exceptions of the ITT analysis (adj HR:1.27; 95%CI: 1.13–1.43), where an increased risk of treatment intensification was observed among DPP4i users.

**TABLE 3:** Risk of treatment intensification in patients using DDP4i compared to those using sulfonylurea: sensitivity analyses.

Analysis	adj HR^a^	[95%CI]
Matching by propensity score	0.93	0.81–1.08
Restriction to patients with “definite” time between first antidiabetic dispensing and index prescription	1.18	0.97–1.43
Start of follow-up time set at 180 days after index prescription	0.96	0.83–1.12
intent-to-treat approach	1.27	1.13–1.43

a Adjusted hazard ratio for all covariates measured at baseline.

## Discussion

In this retrospective cohort study based on administrative healthcare data, the add-on of a DPP4i rather than a SU to MET monotherapy was not associated with a delay of the subsequent treatment intensification. In our cohort of T2D patients, more than half of patients in both treatment groups discontinued the assigned anti-diabetic treatment during follow-up. The observed frequency of discontinuation was consistent with results reported from previous studies (Farr et al., 2014). Side effects, mostly gastrointestinal, and efficacy issues usually represents the main reasons for discontinuation (Farr et al., 2014; Roborel de Climens et al., 2020). In particular, in accordance with the evidences from the literature (Rathmann et al., 2013; Peng et al., 2016; Bloomgarden et al., 2017), in the present study cohort, discontinuation occurred more frequently among patients on MET + SU. A previous retrospective cohort study based on administrative claim-database (Bloomgarden et al., 2017) also found that patients on MET + sitagliptin had both higher adherence and persistence when compared to patients on MET + SU. The known higher risk of hypoglycemic events associated to SU represent a possible explanation for the lower adherence and persistence observed among SU users compared to DPP4i users (Inzucchi et al., 2015b; Valensi et al., 2015; Foroutan et al., 2016). In the present study, however, deviations from the index treatment, like discontinuation or switch, caused the censoring of patients. In particular, this approach allowed controlling for the higher probability of receiving a treatment intensification expected for patients treated with MET + DPP4i compared to those on MET + SU. In fact, due the special reimbursement access criteria applied to DPP4i by the Italian National Healthcare System (Montilla et al., 2014), patients receiving DPP4i are expected to be more strictly monitored than those on SU so that a timely detection of a secondary treatment failure and a consequent treatment intensification is more likely occur.

During the last 2 decades, in many countries, SU have been the most widely used second-line non-insulin hypoglycemic medications (Mishriky et al., 2015; Deacon and Lebovitz, 2016; Foroutan et al., 2016; Moreno Juste et al., 2019). Nevertheless, current guidelines recommend preferring the use of SU as add-on to metformin monotherapy only if costs represent a major issue (Davies et al., 2018). In fact, despite the longer clinical experience available for SU and its comparable hypoglycemic effect with respect to the newer DPP4i, the latter show important advantages in terms of risk of hypoglycemic events and impact on body weight (Inzucchi et al., 2015b; Foroutan et al., 2016).

As for the comparative durability of the hypoglycemic effect of DPP4i vs. SU, instead, current clinical evidences are still poor and inconclusive (Mamza et al., 2016; Chen et al., 2017; Inzucchi et al., 2015a). A meta-analysis of eight double-blind randomized clinical trial reported that MET + DPP4i were associated with significantly smaller increases in the HbA1c level from 24–28–104 weeks compared with MET + SU (mean difference: −0.16%, 95%CI: −0.21 to −0.11; *p* < 0.001). However, on one hand the high rate of lost to follow-up in the included studies threaten results validity while, on the other hand, the clinical relevance of these findings is likely to be negligible (Chen et al., 2017).

Inzucchi et al. (Inzucchi et al., 2015a) conducted a retrospective observational study using a US data source of electronic medical records. The authors compared the time to insulin initiation among T2D patients in a propensity score matched cohort of 3,864 subjects on MET + SU and an equal number of patients on MET + sitagliptin. Findings from this study suggested that patients treated with MET + sitagliptin had a lower risk of insulin initiation compared to those treated with MET + SU (adj HR: 0.761; 95%CI: 0.646–0.897), which become statistically significant after 4 years since study entry. However, exposure misclassification might have biased the results, as the authors could not ascertain if a patient was continuously treated with the index therapy beyond 90 days after enrollment, as required by the study design, or if they discontinued or switched therapy (Inzucchi et al., 2015a). Montivida and others performed an observational retrospective cohort study using the US Centricity Electronic Medical Records stratifying the study population according to the HbA1c levels recorded at time of second-line antidiabetic drug initiation (i.e., HbA1c 7.5–7.9%, 8–9%, 9.1–12%, >12%). The authors reported that patients treated with second-line DPP4i having a baseline HbA1c levels between 7.5% and 12% had slightly higher probability of sustaining glycemic control over 2 years without further intensification than those treated with SU (Montvida et al., 2018). One of the major limitations of this study was the absence of information on treatment adherence during follow-up. Another observational retrospective cohort study from Mamza et al. (Mamza et al., 2016) found that, T2D patients on MET + DPP4i were more likely to experience a substitution or intensification of treatment with a third agent at HbA1c ≥ 7.5% during follow-up compared to those on MET + SU (adjusted HR, 1.58; 95%CI: 1.48–1.68). The inconsistency of results reported by Mamza and others compared to the studies reported above as well as the analyses presented in this paper is likely to be explained by differences in study design and outcome definition. Moreover, as acknowledged by study authors, patients on MET + SU and MET + DPP4i were not required to have comparable persistence or adherence to the treatment during follow-up (Okemah et al., 2018).

### Strengths and Limitations

One of the main strengths of the present study is represented by the emulation of a “per protocol” approach for which deviations from the index treatment like switch and treatment discontinuation caused the censoring of patients from follow-up. As demonstrated by the results of the ITT analysis, this approach allowed limiting the impact of the special reimbursement access criteria applied in Italy to DPP4i, which are expected to favour the timely detection of secondary treatment failure in patients treated with these drugs and, thus, differentially affect the probability of receiving a treatment intensification in the two exposure groups. Moreover, estimates of relative risk were statistically adjusted for several baseline characteristics that can act as confounders. In particular, other than concomitant pharmacotherapies and diabetes-related comorbidities, the time from first metformin dispensing was also included in the model as a proxy of disease duration. Another strength of our study concerns the use of multiple population-based administrative healthcare data sources from four different Italian geographic areas covering about 15% of the whole Italian population. This resulted in a large sample size and a higher generalizability of study findings. However, there are also limitations that should be considered for the correct interpretation of study results. First, the use of administrative healthcare data does not allow to control for clinical characteristics like HbA1c levels, body mass index and physical activity, which are well known risk factors for secondary treatment failure (Kalra et al., 2019). Also, it is noteworthy that secondary treatment failure is actually diagnosed based on periodic HbA1c measurements and that we used the addition of a third non-insulin antidiabetic medication or insulin after at least 180 days following treatment initiation as the study outcome. Although its validity as a proxy of secondary treatment failure was not assessed in the present study, we expect a high positive predictive value, also due to the exclusion of switches to different medications from the outcome definition, which may reflect tolerability rather than efficacy issues (Ekström et al., 2015). Nevertheless, we cannot exclude that a minority of the treatment intensifications observed even after 180 days from treatment initiation were actually primary treatment failures detected with delay. Another study limitation concerns the possible misclassification of exposure. This is intrinsic to the nature of the observational data used for the study. First, dispensing data do not provide information on the actual intake of the dispensed medication. Second, only dispensings of prescription drugs reimbursed by the NHS are captured. Given the chronic nature of diabetes and the fully-reimbursed healthcare assistance provided by the Italian NHS to patients with T2D, exposure misclassification in this study was likely minor and non-differential, although we cannot exclude a possible bias toward the null. Finally, given the observational nature of this study, residual confounder due the differential management and care of patients in the two treatment groups might have possibly affected the results and artefactually increased the risk of treatment intensification for patients on DPP4i relatively to those on SU.

In conclusion, this study found that in patients with T2D from four Italian geographical areas the add-on of a DPP4i rather than a SU to MET monotherapy was not associated with a delay of the subsequent treatment intensification. This study adds further insights to the body of evidence concerning the real-world long-term comparative durability of these two widely used second-line hypoglycemic agents. However, given the limitations related to the observational nature of the study and the heterogeneity of the available clinical evidence, further studies on this topic are warranted to better define the place in therapy and prescribing recommendations for DPP4i with respect to SU, as well as to other available second-line medications for T2D.

## Data Availability

The data analyzed in this study is subject to the following licenses/restrictions: The datasets presented in this article are not readily available because of the privacy legislation. Requests to access the datasets should be directed to the corresponding author. Requests to access these datasets should be directed to giuseppe.roberto@ars.toscana.it.
