# Supplementary material for: Time to Treatment Intensification in Patients Receiving DPP4 Inhibitors Versus Sulfonylureas as the First Add-On to Metformin Monotherapy: A Retrospective Cohort Study
Source: Front Pharmacol. 2022 May 30;13:871052. doi: 10.3389/fphar.2022.871052 (PMC9189773; doi:10.3389/fphar.2022.871052)
Supplement: Supplementary file 1 [file DataSheet1.docx]

Time to treatment intensification in patients receiving DPP4 inhibitors versus sulfonylureas as the first add-on to metformin monotherapy: a retrospective cohort study

Supplementary Material

**Supplementary Table 1**. Distribution of the study population according to DD4i or SU use and geographical area.

|  | **MET+DDP4i**  **(6261)** | **MET+SU**  **(8673)** | **Total**  **(14.934)** |
| --- | --- | --- | --- |
|  | **N (%)** | **N (%)** | **N (%)** |
| **Tuscany** | 2434 (38.9) | 4159 (47.9) | 6593 (44.1) |
| **Piedmont** | 1993 (31.8) | 2895 (33.4) | 4888 (32.7) |
| **Umbria** | 1007 (16.1) | 696 (8.0) | 1703 (11.4) |
| **Caserta** | 827 (13.2) | 923 (10.6) | 1750 (11.7) |

MET = metformin; DDP4i = [dipeptidyl peptidase inhibitor](https://www.google.com/search?rlz=1C1GCEV_en&q=dipeptidyl+peptidase+inhibitor&spell=1&sa=X&ved=0ahUKEwiuq57NyvXhAhVOalAKHcmbDd0QkeECCCooAA); SU = sulfonylurea

**Supplementary Table 2**. Frequency of censoring events by type.

|  | **DPP4i** | **SU** |
| --- | --- | --- |
|  | **N=5300 (100%)** | **N=5300 (100%)** |
| Mean follow-up time | 1.9 | 1.2 |
| *First reason for censoring* |  |  |
| Index Drug or MET Discontinuation | 5343 (66.8%) | 4056 (76.5%) |
| Index Drug Discontinuation | 2859 (53.9%) | 3165 (59.7%) |
| MET Discontinuation | 684 (12.9%) | 891 (16.8%) |
| Treatment Intensification | 491 (9.3%) | 272 (5.1%) |
| TI within 180 from index prescription | 419 (7.9%) | 506 (9.5%) |
| Switch | 371 (7.0%) | 223 (4.2%) |
| End of Study Period | 202 (3.8%) | 67 (1.3%) |
| Cancer | 172 (3.2%) | 141 (2.7%) |
| Add-on with only 1 dispensation* | 102 (1.9%) | 35 (0.7%) |

DDP4i = [dipeptidyl peptidase inhibitor](https://www.google.com/search?rlz=1C1GCEV_en&q=dipeptidyl+peptidase+inhibitor&spell=1&sa=X&ved=0ahUKEwiuq57NyvXhAhVOalAKHcmbDd0QkeECCCooAA); SU = sulfonylurea; MET = metformin; TI = treatment intensification

*no second dispensing registered for the third non-insulin antidiabetic drug

**Supplementary Figure 1**. Study design


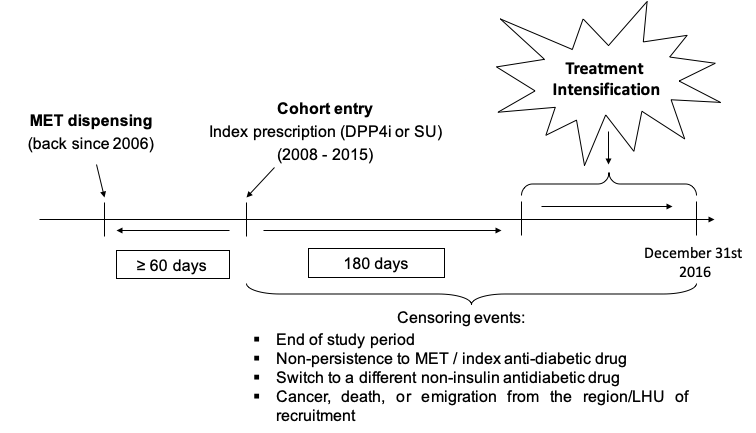


***Appendices***

**Appendix 1**. Antidiabetic drugs available in Italy during the study period

| **Pharmacologic class** | **ATC** | **Active substance** |
| --- | --- | --- |
| DDP-4 inhibitor | A10BH01 | sitagliptin |
|  | A10BH02 | vildagliptin |
|  | A10BH03 | saxagliptin |
|  | A10BH04 | alogliptin |
|  | A10BH05 | linagliptin |
|  | A10BD07 | metformin and sitagliptin |
|  | A10BD08 | metformin and vildagliptin |
|  | A10BD09 | pioglitazone and alogliptin |
|  | A10BD10 | metformin and saxagliptin |
|  | A10BD11 | metformin and linagliptin |
|  | A10BD13 | metformin and alogliptin |
| GLP-1 analogues# | A10BJ01 | exenatide |
|  | A10BJ02 | liraglutide |
|  | A10BJ03 | lixisenatide |
|  | A10BJ04 | albiglutide |
|  | A10BJ05 | dulaglutide |
| Biguanides | A10BA01 | fenformin |
|  | A10BA02 | metformin |
| Sulfonylureas | A10BB01 | glibenclamide |
|  | A10BB02 | chlorpropamide |
|  | A10BB03 | tolbutamide |
|  | A10BB06 | carbutamide |
|  | A10BB07 | glipizide |
|  | A10BB08 | gliquidone |
|  | A10BB09 | gliclazide |
|  | A10BB12 | glimepiride |
| Thiazolidinediones | A10BG02 | rosiglitazone |
|  | A10BG03 | pioglitazone |
|  | A10BD04 | glimepiride rosiglitazone |
|  | A10BD06 | glimepiride pioglitazone |
| Alfa glicosidase inhibitors | A10BF01 | acarbose |
| Meglitinides | A10BX02 | repaglinide |
| Insulins | A10A* | insulin and analogues |
| Other hypoglicemic drugs in fixed combinations | A10BD01 | phenformin and sulfonamides |
|  | A10BD02 | metformin and sulfonamides |
|  | A10BD03 | metformin and rosiglitazone |
|  | A10BD05 | glimepiride and pioglitazone |
|  | A10BD14 | metformin and repaglinide |
| SGLT2 inhibitors# | A10BK01 | dapaglifozin |
|  | A10BK02 | canaglifozin |
|  | A10BK03 | empaglifozin |
|  | A10BD15 | metformin dapagliflozin |
|  | A10BD16 | metformin canagliflozin |
|  | A10BD20 | metformin empagliflozin |

DPP4= dipeptidyl peptidase-4; GLP-1 = Glucagon-like peptide 1; SGLT2 = Sodium-glucose co-transporter 2 #ATC/DDD alterations 2017 (https://www.whocc.no/atc_ddd_index/updates_included_in_the_atc_ddd_index/atc_ddd_alterations_2017/)

**Appendix 2**. Start of data availability

| **Registry** | **Piedmont** | **Tuscany** | **Umbria** | **Caserta** |
| --- | --- | --- | --- | --- |
| Inhabitant registry | 2000 | 2004 | 2000 | 2000 |
| Hospital discharge records | 2011 | 1997 | 2000 | 2006 |
| Drug registry | 2012 | 2003 | 2011 | 2006 |
| Exemption from copayment registry | 2000 | 1999 | NA | 2006 |
| Registry of utilization of secondary care encounters and diagnostic procedures | 2011 | 2002 | NA | 2009 |

NA: not available

**Appendix 3**. Operational definitions of persistence, insulin initiation, add-on and switch

*Persistence*

It was defined as the absence of a gap ≥90 days between two treatment episodes. Each dispensing of the drug of interest corresponded to a treatment episode (see Figure below). The duration of each treatment episode (i.e. days covered by the medication) was estimated as the ratio between the total amount of active principle dispensed and the corresponding Defined Daily Dose ([www.whocc.no/atc_ddd_index](http://www.whocc.no/atc_ddd_index)). The end of a treatment episode corresponded to either: i) the date of dispensing plus the estimated duration, or ii) the date of the subsequent dispensing for the prescription refill (i.e. no stockpiling will be allowed).

Two examples of patients that are respectively *persistent* (A) and *non-persistent* (B) to the medication of interest.

*Insulin initiation*

It must occur before discontinuation (see the definition of persistence) of anyone of the two hypoglicemic agents corresponding to the index dual therapy. For the primary analysis, the event date is the date of first insulin dispensing, while for the sensitivity analysis the event date is be the date of second insulin dispensing occurred without insulin discontinuation between the first and the second dispensing (the other drugs may also be discontinued after the first insulin).

In case discontinuation of insulin occurred before the second insulin dispensing, the patients were censored at the time of insulin discontinuation (i.e. day 90 after the end of the duration of the first insulin dispensing).

*Add-on of the third non-insulin antidiabetic drug*

It must occur before discontinuation (see the definition of persistence) of anyone of the two hypoglicemic agents corresponding to the index dual therapy. There must be a second dispensing for all the three drugs after the first dispensing of the third hypoglicemic agent. The latest of these dispensings corresponded to the event date.

*Switch to third non-insulin antidiabetic drug*

It occurs when a third non-insulin antidiabetic agent was dispensed before discontinuation (see the definition of persistence) of anyone of the two hypoglicemic agents corresponding to the index dual therapy. The date of second dispensing of the third non-insulin antidiabetic drug is the event date. At this date, anyone of the two hypoglicemic agents corresponding to the index dual therapy must be discontinued (see the definition of persistence).

**Appendix 4**. Time between first antidiabetic drug to index dispensing

Two examples of patients for which the time between 1^st^ antidiabetic drug and index dispensing will be respectively classified as “definite” (A) or “uncertain” (B).

**

**Appendix 5**. Hospitalization for diabetes complications or other comorbidities^1^

| **Complications or other comorbidities** | | **ICD-9 code** |
| --- | --- | --- |
| Cardiovascular diseases | Acute myocardial infarction | 410 |
|  | Acute ischemic heart disease | 411 |
|  | Angina pectoris | 413 |
|  | Operations on vessels of heart | Procedure code: 36 |
| Cerebrovascular diseases | Cerebrovascular diseases | 430-436 |
| Retinopathy | Retinopathy | 362.0, 362.1, 362.2 |
|  | Diabetes with ophthalmic manifestations | 250.5 |
| Nephropathy | nephropathy | 583 |
|  | Diabetes with renal manifestations | 250.4 |
|  | Acute kidney failure | 584 |
| Neuropathy | Peripheral neuropathy | 356 |
|  | Diabetes with neurological manifestations | 250.6 |
| Peripheral vascular disorders | Diabetes with peripheral circulatory disorders | 250.7 |
|  | Amputation of lower limb | Procedure code: 84.11-84.19 |
|  | Ulcer of lower limbs, except pressure ulcer | 707.1 |
|  | Peripheral angiopathy | 443.81 |

^1^Diagnoses either in primary or secondary position recorded during 1 year before index prescription.

**Appendix 6**. Pharmacotherapies measured at baseline

| **Pharmacotherapy*** | **ATC code** |
| --- | --- |
| Antidepressants | N06A |
| Corticosteroids for systemic use | H02 |
| Lipid lowering drugs | C10 |
| Anticoagulants | B01, excl B01AC |
| Antiplatelets | B01AC |
| Beta blockers | C07 |
| Antihypertensives and/or diuretics | C02-C03 |
| Dihydropyridine CCB | C08CA |
| Non Dihydropyridine CCB | C08D |
| Angiotensin receptor blockers and ACE-I | C09 |
| Antipsychotics | N05A |

*≥2 dispensings during 1 year before index prescription

ICD: international classification of diseases; ATC: anatomic therapeutic chemical; CCB: calcium channel blocker; ACE-I: angiotensin converting enzyme inhibitor;
